# Lifestyle Risk Factors for Breast Cancer in BRCA1/2-Mutation Carriers Around Childbearing Age

**DOI:** 10.1007/s10897-016-0049-4

**Published:** 2016-12-13

**Authors:** A. van Erkelens, L. Derks, A. S. Sie, L. Egbers, G. Woldringh, J. B. Prins, P. Manders, N. Hoogerbrugge

**Affiliations:** 1 Department of Human Genetics 836, Radboud University Medical Center, PO BOX 9101, 6500 HB Nijmegen, The Netherlands; 2 Department of Medical Psychology, Radboud University Medical Center, Nijmegen, the Netherlands

**Keywords:** *BRCA*, Lifestyle, Risk factor, Breast cancer, Physical activity, Overweight, Children

## Abstract

BRCA1/2-mutation carriers are at high risk of breast cancer (BC) and ovarian cancer. Physical inactivity, overweight (body mass index ≥25, BMI), smoking, and alcohol consumption are jointly responsible for about 1 in 4 postmenopausal BC cases in the general population. Limited evidence suggests physical activity also increases BC risk in BRCA1/2-mutation carriers. Women who have children often reduce physical activity and have weight gain, which increases BC risk. We assessed aforementioned lifestyle factors in a cohort of 268 *BRCA1/2*-mutation carriers around childbearing age (born between 1968 and 1983, median age 33 years, range 21–44). Furthermore, we evaluated the effect of having children on physical inactivity and overweight. Carriers were asked about lifestyle 4–6 weeks after genetic diagnosis at the Familial Cancer Clinic Nijmegen. Physical inactivity was defined as sports activity fewer than once a week. Carriers were categorized according to the age of their youngest child (no children, age 0–3 years and ≥4 years). In total, 48% of carriers were physically inactive, 41% were overweight, 27% smoked, and 70% consumed alcohol (3% ≥8 beverages/week). Physical inactivity was 4–5 times more likely in carriers with children. Overweight was not associated with having children. Carriers with children are a subgroup that may specifically benefit from lifestyle support to reduce BC risk.

## Introduction

Less than 10% of all breast cancer (BC) cases is caused by a genetic predisposition, most commonly due to a mutation in one of the *BRCA* -genes. Female *BRCA1/2*-mutation carriers have a cumulative lifetime risk of BC up to 60–80%, and an increased risk of ovarian cancer (OC) up to 20–60% for *BRCA1* and 5–20% for *BRCA*2 (Antoniou et al. 2003; Chen and Parmigiani 2007). Age at onset of BC is younger for hereditary tumors compared to sporadic tumors (42 versus 53 years, respectively) (van der Kolk et al. 2010). Differences in penetrance between generations suggest that BC risk in *BRCA1/2*-mutation carriers is also influenced by environmental and lifestyle factors (Narod 2006).

To date, especially physical inactivity below age 30 is associated with an increased BC risk in *BRCA1/2*-mutation carriers (Pijpe et al. 2010). Less is known about the risks of smoking, alcohol intake, adult weight gain and overweight on BC risk in *BRCA1/2*-mutation carriers (Friebel et al. 2014; Pettapiece-Phillips et al. 2015). In the general population, drinking alcoholic beverages (Chen et al. 2011), smoking (Gaudet et al. 2013), and physical inactivity (Chlebowski 2013; Wu et al. 2013) are well known lifestyle risk factors for pre- and postmenopausal BC. High body weight and adult weight gain increase the risk of postmenopausal BC (Friedenreich 2001). One in four BC cases in the general population may be attributed to lifestyle factors (van Gemert et al. 2015).

In the general population, women who have children often decrease physical activity and have weight gain (Engberg et al. 2012). Among *BRCA1/2*-mutation carriers, a similar effect of having children is possible. Because physical inactivity is a likely modifier of BC risk in BRCA1/2-mutation carriers, it is important to know whether *BRCA1/2*-mutation carriers around childbearing age are a subgroup that may specifically benefit from lifestyle support. The aims of our study were to assess the prevalence of physical inactivity, overweight, smoking, and alcohol consumption in a cohort of BRCA1/2-mutation carriers around childbearing age, and to evaluate the association between physical inactivity, overweight and having children.

## Methods

### Participants

The current study sample was a subset from a cohort of female *BRCA1/2-*mutation carriers who were asked about lifestyle factors in person by their physicians (NH and GW) during intake, on average four to six weeks after *BRCA1/2-*diagnosis. All consultations took place at the Familial Cancer Clinic of the Radboud university medical center between 1996 and 2012. *BRCA1/2*-mutation carriers born between 1968 and 1983 were selected for this study because they were considered being around childbearing age (i.e. most likely to have either no or young children). Women were classified as having no children or according to the age of their youngest child (0–3 years and ≥4 years). This subdivision was based on the age of compulsory education in the Netherlands beginning at the age of four year. Women who had a stillbirth, miscarriage, were pregnant at time of, or gave birth after the first consultation were classified as having no children. Data from patients’ (electronic) medical records were collected and assessed. This study was found to be exempt from review by the Radboud university medical center institutional ethics board.

### Instrumentation

Clinical characteristics noted in the medical records regarding *BRCA1/2*-diagnosis (type, year, and age at diagnosis), personal history of BC, parity and lifestyle characteristics were collected by NH and GW using a standard questionnaire during the first consultation. Primary outcomes were physical inactivity and Body Mass Index (BMI). Being physically inactive was stated yes if a woman did not participate in sports activities (e.g. fitness, cycling, jogging, tennis, etc.) at least once a week. No further quantification or subdivision based on a more detailed type or frequency of sports activity could be made due to the limited amount of data. Body weight at the first consultation and height were used to calculate BMI. A BMI ≥25 kg/m^2^ was used as the cut-off value for being overweight (AICR/WCRF 2007). Secondary outcomes were number of alcoholic beverages consumed per week and current smoking habits. A low-risk lifestyle for BC was defined as being physically active, not being overweight, and not smoking, in addition to drinking a maximum number of seven alcoholic beverages/week (AICR/WCRF 2007).

### Data Analysis

Patient characteristics and measures were analyzed using descriptive statistics. All data are presented as median with range or counts and percentages where appropriate. A Kruskall-Wallis test for non-normal distributed variables was used to compare BMI score, number of alcoholic beverages consumed/week, and age. A Chi-square test, or a Fisher exact test if appropriate, was performed to compare overweight (BMI ≥ 25) and alcohol consumption (0, 1–7 and ≥8 beverages/week), current smoking habits (yes / no), physical inactivity and BC prevalence. Lastly, a Mann-Whitney U test for non-normal distributed variables was used to compare age at first childbirth and number of children between parous women.

A comparison was made between inactive/active and not overweight/overweight *BRCA1/2*-mutation carriers using either a Mann-Whitney U test (age at *BRCA1/2*-diagnosis, BMI score, and number of alcoholic beverages/week) or a Chi Square, or Fisher exact test if appropriate (overweight, alcoholic consumption [0, 1–7 and ≥8 beverages/week], smoking habits, and physical inactivity). In multivariate logistic regression analysis, the relation with physical inactivity and overweight was examined for all factors that were analyzed in univariate analyses, with stepwise removal of non-significant factors. Two sided *p*-values below 0.05 were considered statistically significant. All computations were performed using SPSS 20.0.

## Results

Data were collected from a total of 270 *BRCA1/2-*mutation carriers. Two carriers who had 70% of the relevant data missing were excluded from statistical analysis; therefore, 268 *BRCA1/2-*mutation carriers were included for further analysis.

Characteristics of the entire cohort and after clustering according to the categorized age of the youngest child are shown in Table 1. There were 165 patients with a *BRCA1/21*-mutation (62%) and 102 with a *BRCA*2-mutation (38%); one patient had a *BRCA1* as well as a *BRCA2*-mutation. Median age at *BRCA1/2* diagnosis was 33 years [range 21–44]. This was higher for women with children compared to women without children (0-3y: 33 years [range 25–43] and ≥4y: 37 years [range 24–43] versus no children: 29 years [range 21–44], *p* < 0.001). No difference was found in number of children. Thirty-four *BRCA1/2*-mutation carriers (13%) were diagnosed with BC prior to attending the Family Cancer Clinic (median age of BC diagnosis 33 years [range 25–43]), however, no difference was found in lifestyle factors between affected and healthy women.

**Table 1:** Characteristics of cohort *BRCA1/2*-mutation carriers, clustered according to age of youngest child. Median [range] or N (%); 1996–2012

			Subdivision based on age of the youngest child						
Characteristics	Total cohort (*N* = 268)*		No children (*N* = 116)*		Youngest child age 0–3 years (*N* = 87)*		Youngest child age ≥ 4 years (*N* = 64)*		*P*-value
Age at *BRCA*-diagnosis (years)	33	[21–44]	29	[21–44]	33	[25–43]	37	[24–43]	< 0.001^c^
*BRCA-*mutation									0.262^b^
*BRCA1*	165	(62)	71	(62)	60	(69)	34	(53)	
*BRCA2*	102	(38)	44	(38)	27	(31)	30	(47)	
Breast cancer diagnosis									0.125^b^
Yes	34	(13)	12	(10)	9	(10)	13	(20)	
No	230	(87)	104	(90)	75	(90)	51	(80)	
BMI (kg/m^2^)									
Median	23.9	[16.9–42.0]	23.2	[16.9–40.5]	24.7	[18.5–42.0]	24.3	[17.1–39.9]	0.060^c^
< 25	143	(59)	69	(67)	44	(56)	30	(52)	0.043^b^
≥ 25	98	(41)	34	(33)	35	(44)	28	(48)	
Physically inactive									0.003^b^
Yes	126	(48)	43	(37)	53	(62)	31	(48)	
No	139	(52)	72	(63)	33	(38)	33	(52)	
Currently smoking									0.014^b^
Yes	72	(27)	39	(34)	14	(16)	19	(30)	
No	195	(73)	77	(66)	73	(84)	34	(70)	
Alcoholic beverages / week									
Median	1	[0–21]	2	[0–21]	1	[1–14]	1	[1–21]	0.061^c^
0	79	(30)	27	(23)	29	(33)	23	(61)	0.183^b^
1–7	177	(66)	83	(72)	54	(62)	39	(36)	
≥ 8	9	(3)	5	(4)	2	(2)	2	(3)	

*Numbers do not always add up due to missing values. One patient with no children had a both a *BRCA*1 and *BRCA*2-mutation

^b^Chi-square test or Fisher exact test

^c^Kruskall-Wallis

^d^Mann-Whitney U test

Forty-eight percent of *BRCA1/2*-mutation carriers were physically inactive, while 41% were overweight, 27% currently smoked, and 3% drank eight or more alcoholic beverages per week. Women with young children (0-3y) were more likely to be physically inactive compared to women with older (≥4y) or no children (62% versus 48% and 37%, respectively, *p* = 0.003). Furthermore, fewer women with young children (0-3y) currently smoked, compared to those with older (≥4y) and no children (16% versus 30% and 34%, respectively, *p* = 0.014). Both women with younger (0-3y) and older (≥4y) children were more often overweight than those with no children (44% and 48% versus 33%, respectively, *p* = 0.043).

Predictors of physical inactivity and overweight in *BRCA1/2*-mutation carriers are shown in Tables 2 and 3. After multivariate logistic regression analysis, physical inactivity was 3–5 times more likely in carriers with children compared to carriers without children (0-3y: OR = 5.03, 95%CI = 2.45–10.33 and ≥4y: OR = 3.54, 95%CI = 1.48–8.45, respectively). Smokers were more than two times more likely to be physically inactive than non-smokers (OR = 2.07, 95%CI = 1.10–3.88). Age at *BRCA1/2*-diagnosis as a continuous variable was associated with a decrease in physical inactivity (OR = 0.93/year, 95%CI = 0.86–0.99) and an increase in overweight prevalence (OR = 1.07/year, 95%CI = 1.02–1.13).

**Table 2:** (final model): Logistic regression model predicting likelihood of physical inactivity in a cohort of *BRCA1/2*-mutation carriers around childbearing age

Variable	*R* ^*2*^ *	*AIC*	*B*	*SE*	*z*	*p*	OR [95% CI]
Likelihood of physical inactivity^1^	0.135	311.4					
Current smoking			0.727	0.321	2.263	0.024	2.07 [1.10–3.88]
Youngest child age 0-3y			1.615	0.367	4.418	<0.001	5.03 [2.45–10.33]
Youngest child age ≥ 4y			1.263	0.444	4.394	0.004	3.54 [1.48–8.45]
Age			-0.078	0.035	2.201	0.027	0.93/year [0.86–0.99]
Likelihood of overweight (BMI ≥ 25)^2^	0.041	317.7					
Age			0.071	0.901	2.65	0.002	1.07/year [1.02–1.13]

Backward Wald. ^1^Included but not in the final model: Personal history of BC, alcohol consumption (beverages/week) and categorized BMI (<25 and ≥25 kg/m^2^). ^2^Included but not in the final model: Personal history of BC, current smoking, alcohol consumption (beverages/week), age of the youngest child (0-3y ≥ y versus no children), and physical inactivity (yes and no). All variables known for *N* = 235. Physically inactive *N* = 123. * Nagelkerke’s R^2^. *AIC* Akaike information criterion; *SE* standard error; *OR* odds ratio. All variables known for *N* = 235. Physically inactive *N* = 123. * Nagelkerke’s R^2^

**Table 3:** (initial model): Logistic regression model predicting likelihood of physical inactivity in a cohort of *BRCA1/2*-mutation carriers around childbearing age

Variable	*R* ^*2*^ *	*AIC*	*B*	*SE*	*z*	*p*	OR [95% CI]
Likelihood of physical inactivity	0.162	311.924					
Current smoking			0.742	0.324	2.29	0.022	2.10 [1.11–3.960]
Youngest child age 0-3y			1.668	1.375	4.449	<0.001	5.30 [2.54–11.06]
Youngest child age ≥ 4y			1.257	0.450	7.822	0.005	3.52 [1.46–8.49]
Age			-0.093	0.037	2.50	0.012	0.91/year [0.85–0.98]
Overweight			0.222	0.287	0.774	0.439	1.25 [0.71–2.19]
Personal history of breast cancer			-0.631	0.458	0.378	0.168	1.88 [0.77–4.61]
Alcoholic beverages / week			-0.006	0.009	0.597	0.550	0.994 [0.987–1.024]
Likelihood of overweight (BMI ≥ 25)	0.051	325.909					
Current smoking			-0.71	0.318	0.223	0.824	0.93 [0.50–1.74]
Youngest child age 0-3y			0.228	0.356	0.641	0.521	1.26 [0.63–2.52]
Youngest child age ≥ 4y			0.157	0.418	0.375	0.707	1.17 [0.52–2.66]
Age			0.061	0.034	1.775	0.076	1.06/year [0.99–1.14]
Physically inactive			0.230	0.288	0.800	0.424	0.26 [0.72–2.21]
Personal history of breast cancer			0.169	0.427	0.394	0.0.693	1.18 [0.51–2.74]
Alcoholic beverages / week			0.000	0.001	0.257	0.798	1.00 [0.998–1.003]

Backward Wald. All variables known for *N* = 235. Physically inactive *N* = 123. * Nagelkerke’s R^2^. *AIC*: Akaike information criterion; *SE*: standard error; *OR *: odds ratio

The number of cumulative high-risk lifestyle factors is shown in Table 4. Data on all lifestyle factors were known for 237 *BRCA1/2*-mutation carriers. Almost four out of ten women had at least two high-risk lifestyle factors (38%), while 6 % had at least three, and one patient had four high-risk lifestyle factors. Twenty-six percent was categorized as having a low-risk lifestyle for BC (physically active, not overweight, not smoking, drinking fewer than 8 alcoholic beverages/week).

**Table 4:** *BRCA1/2-*mutation carriers with one or more high-risk lifestyle factors* (*n* = 237)

Number of high-risk lifestyle factors	N	%
0	61	(26)
1	87	(37)
2	28	(32)
3	14	(6)

*All combinations possible: smoking (yes/no); BMI ≥ 25 (yes/no); physically inactive (yes/no); number of alcoholic beverages consumed (<8 or ≥8/week)

## Discussion

Our data show that nearly half of *BRCA1/2*-mutation carriers around childbearing age (48%) were physically inactive, while 41% were overweight, 27% currently smoked and 70% consumed alcoholic drinks (3% eight or more beverages a week). Four out of ten *BRCA1/2-*mutation carriers (38%) had at least two high-risk lifestyle factors for BC. Physical inactivity was 4–5 times more likely in carriers who had children compared to carriers without children (0-3y: OR = 5.03, 95%CI = 2.45–10.33, and ≥4y: OR = 3.54, 95%CI = 1.48–8.45), and two times more likely in current smokers (OR = 2.07, 95%CI = 1.10–3.88). This is consistent with literature showing that having children is associated with decreased physical activity levels in women (Engberg et al. 2012). Physical inactivity decreased with age (OR = 0.93/year, 95%CI = 0.86–0.99) while overweight prevalence increased with age (OR = 1.07/year, 95%CI = 1.02–1.13). Carriers with young children (0-3y) were less likely to smoke compared to carriers with older (≥4y) or no children (16% versus 30% and 34%, *p* = 0.014). No other associations between having children and lifestyle factors were observed.

Previous literature shows that women with a family history of BC do not practice a healthier lifestyle than women without a family history (Townsend et al. 2013). McLeish et al. describes that women with a family history of BC are willing to change their lifestyle risk factors, and that 35% of women reported increasing their physical exercise since learning of their familial BC risk (McLeish et al. 2013). Furthermore, younger women (<40 years) had the least healthy lifestyle, and made the fewest lifestyle changes. A study by O’Neill et al. describes no changes in diet and physical activity six months after disclosure of *BRCA1/2* test results (O'Neill et al. 2008). The lifestyle our study population has is similar to that of the Dutch population (overweight 48%, smoking 23%, physically inactive 37% (CBS 2014)).

An increasing body of observational evidence underlines the importance of a healthy lifestyle for non-hereditary BC incidence (Chen et al. 2011; Chlebowski 2013; Friedenreich 2001; Gaudet et al. 2013; Wu et al. 2013) and outcome (Davies et al. 2011), as well as for the prevention of secondary malignancies in cancer survivors (Travis et al. 2013). Literature suggests that exercise training may be beneficial for health-related quality of life during active cancer treatment (Mishra et al. 2012) and may improve aerobic exercise tolerability in sedentary cancer survivors (Bourke et al. 2014). One of the mechanisms by which diet and physical activity may improve BC outcome is by decreasing excess body weight (Davies et al. 2011). Obesity is associated with a poorer prognosis of non-hereditary BC (Carmichael 2006) and may further increase the risk of unwanted treatment effects like poorer outcomes after breast surgery and decreased quality of life (Demark-Wahnefried et al. 2012).

### Practice Implications

Physical activity below age 30 has been reported to be protective for BC in *BRCA1/2*-mutation carriers (Pijpe et al. 2010). It is less clear whether other lifestyle factors (smoking, alcohol consumption, overweight) affect BC risk in *BRCA1/2*-mutation carriers like in the general population (Friebel et al. 2014; Pettapiece-Phillips et al. 2015). *BRCA1/2*-mutation carriers with children and those who smoke may represent a potential subgroup for which additional health benefits might be achieved.

Under-recognition of modifiable lifestyle risk factors for BC by both *BRCA*-mutation carriers and doctors may be a significant barrier to promotion of a healthy lifestyle (Albada et al. 2014). *BRCA1/2*-mutation carriers tend to perceive mainly external factors to be related to their health (Thomson et al. 2014). A recent study showed that genetic counselors provided brief, general lifestyle information to only 19% of counselees and individual lifestyle advice to only 6% (Albada et al. 2014).

Changing lifestyle behavior starts with increasing awareness, which requires consistent, personalized lifestyle modification counseling by counselors (Kreuter et al. 2000; Lin et al. 2014). Physicians can offer more support by adequately noting changes in modifiable factors such as body weight and physical inactivity and give proper advice regarding these lifestyle factors during surveillance visits. Motivational interviewing can be used during consultations to help carriers become active participants in talking about lifestyle change (Albada et al. 2014; Rollnick et al. 2010). Furthermore, these visits offer an opportunity for education and development of a personalized action plan regarding lifestyle behaviors. Obese carriers who want to change health behavior may be referred to a nutritionist or bariatric specialist for weight management, and current smokers may be referred to quit smoking programs, all of which are covered by mandatory health insurance in the Netherlands. Physical activity may be promoted by consultation with a sports therapist.

### Study Limitations

The present study has some strong and weak points that should be considered in the interpretation of the results. The primary strength of our study is that lifestyle was measured on average four to six weeks after *BRCA1/2-*mutation diagnosis when their *BRCA1/2-*diagnosis was unlikely to affect their lifestyle. The main limitation of our study is that we measured lifestyle factors only once and only, which gives a snapshot that might not be representative. Furthermore, no subdivisions could be made based on frequency or intensity of physical exercise, which limits criterion validity. Because of a broad definition of sports activities, it is likely that we have underestimated physical inactivity in our study. If so, the prevalence of physical inactivity might be even higher than reported here. Our analysis only included women born from 1968 to 1983 with a median age of 33 years [range 21–44], which limits generalization to older and younger *BRCA1/2*-mutation carriers. Different physicians collected information (NH and GW), and data were self-reported and thus may have included socially desirable answers. Lastly, current employment status and educational level were not documented in the medical record, while both are known to be reflective of social economic status and have an important bearing on daily activity levels (Adler and Ostrove 1999; Hanson and Chen 2007): future studies should take these potential confounders into account, and should assess lifestyle factors at multiple time points.

### Research Recommendations

More studies are needed to assess whether lifestyle affects BC risk in *BRCA1/2-*mutation carriers like in the general population, whether lifestyle interventions can reduce this risk, and how to best implement sustainable lifestyle changes (diet, exercise, weight) (Eccles et al. 2013)*.* Several prospective trials are currently ongoing to assess the feasibility of lifestyle changing interventions to reduce BC risk in high-risk populations (Kiechle 2014; Reding 2013). Besides modifying BC risk, promotion of a healthy lifestyle may also improve risk factors for cardiovascular disease (CVD) (Lin et al. 2014). This may be important, as *BRCA1/2*-mutation carriers are potentially at increased CVD risk because of early menopause around age 40 (due to risk-reducing salpingo-oophorectomy), or cardiotoxic effects after adjuvant breast cancer treatment (Arts-de Jong et al. 2014).

## Conclusion

In BRCA1/2-mutation carriers around childbearing age, 48% was physically inactive, 41% overweight, 27% smoked, and 70% consumed alcohol (3% ≥8 alcoholic beverages/week). Physical inactivity was 4–5 times more likely in carriers with children and smokers. Overweight was not associated with having children. Because physical inactivity is a likely modifier of breast cancer risk in BRCA1/2-mutation carriers, carriers with children are a subgroup that may specifically benefit from lifestyle support to reduce BC risk.
